# A Pre-Screening Tool to Assess Dog Suitability for Animal-Assisted Interventions: Preliminary Results for Dog-Suitability Tests (*SuiTe*)

**DOI:** 10.3390/vetsci12121110

**Published:** 2025-11-22

**Authors:** Giulia Russo, Carmen Borrelli, Giacomo Riggio, Elisa Rosson, Matilde Bentivoglio, Chiara Mariti

**Affiliations:** 1 Department of Veterinary Sciences, University of Pisa, 56124 Pisa, Italy; carmen.borrelli@phd.unipi.it (C.B.); giacomoriggio@gmail.com (G.R.); e.rosson@studenti.unipi.it (E.R.); m.bentivoglio1@studenti.unipi.it (M.B.); chiara.mariti@unipi.it (C.M.); 2 Vethos-Etologia Clinica Veterinaria, 00182 Rome, Italy

**Keywords:** animal-assisted interventions, animal-assisted services, visiting dogs, therapy dogs, dog suitability, behavioural test, aptitude test, questionnaire, valence of behaviour

## Abstract

Animal-assisted interventions (AAIs) or Services (AAS) may cause stress in participating dogs, making the selection of suitable individuals essential to prevent strain. Different non-standardized approaches currently exist to assess dogs’ suitability for AAIs. This preliminary study aimed at evaluating two combined tools, a behavioural aptitude test (*SuiTe*) and an ad hoc revised questionnaire incorporating C-BARQ, for pre-screening dog suitability for AAIs, also in relation to salivary cortisol measured by enzyme immunoassay in *N* = 38 dogs. Dogs’ behavioural responses to environmental and social stimuli were scored on an X-Y scale and classified by two independent evaluators as suitable (S), pending suitability (P), or unsuitable (U). Non-parametric tests were performed (*p* < 0.05). Results indicated significant differences between dogs classified as S or P versus U, both in *SuiTe* valence scores (higher in S and P) and in separation, attachment, and fear/anxiety behaviours assessed by the questionnaire (higher in U). However, suitability in the *SuiTe* was lower than that assessed by caregivers through an open question. Our study highlights the complexity of this assessment and the limited awareness of caregivers regarding the situations their dogs face every day. Future analyses will refine this multiparametric approach within a One Welfare perspective, ensuring the welfare of both animals and humans involved in AAIs.

## Simple Summary

The increasing popularity of animal-assisted interventions (AAIs) or services (AAS) reflects their impact on promoting human health and well-being, while emphasizing the importance of ensuring the welfare of the participating animals. In this study, a protocol was developed to preliminarily assess dog suitability for participation in AAIs. This type of assessment, focused on dog behaviour and combined with a questionnaire and salivary cortisol measurement, presents several complexities. Preliminary results showed that the positive or negative valence (i.e., the positive or negative character of an emotion) of behaviours distinguishes suitable dogs from unsuitable ones, and that differences emerge in behaviours reflecting the relationship with the owner as well as fear or anxiety. Moreover, dog owners may not always be able to recognize whether their dogs are suitable or not. These findings could also be valuable for dogs not involved in AAIs, as they may reveal how dogs behave in everyday situations and help owners ensure their welfare.

## 1. Introduction

Animal-assisted interventions (AAIs), or animal-assisted services (AAS) to use new uniform terminology at the international level [1], is the umbrella term for structured human–animal interactions with a therapeutic, rehabilitative, educational, and recreational purpose, that involves the engagement of domestic animals to improve the health and well-being of people with various physical, neuromotor, mental, and psychological disorders [2]. These kinds of activities can even involve healthy people to improve their social or relational skills, their knowledge or awareness around a topic, or to stimulate participation and motivation [2]. AAIs can be divided, depending upon who is delivering the service, the intended outcomes, and how it is delivered, into three categories:

- Animal-assisted therapy (AAT) [2] or animal-assisted treatment AATx [1]: A therapeutic intervention/treatment aimed at addressing and managing disorders affecting physical, neuropsychomotor, cognitive, emotional, and relational domains, intended for individuals with physical, psychological, sensory, or multiple conditions. These kinds of interventions are usually intended for an individual or a very small group and are tailored to the users. According to the Italian regulatory framework, AAT involves the designation of two professional figures: a medical specialist or psychologist–psychotherapist, responsible for defining the project objectives, and a qualified healthcare professional, responsible for managing the patient during the sessions.
- Animal-assisted education (AAE) [2]: An education-oriented intervention designed to promote, activate, and support individuals’ internal resources and growth potential, fostering their capacity for personal development, relational engagement, and social inclusion. This approach can be delivered individually or in group settings and aims to enhance overall well-being within the person’s living environment, especially in institutional contexts where adaptive skills are required. AAEs also facilitate behavioural re-education processes, contributing to improved quality of life and strengthening self-esteem in participants. AAE interventions are applicable in a wide range of contexts.
- Animal-assisted activity (AAA) [2] or animal-assisted support programmes (AASP) [1]: Interventions with recreational, leisure, and socialization purposes, designed to enhance quality of life and foster appropriate human–animal interactions. These activities do not include competitive or sporting engagements involving animals. In AAA, the interaction with the animal serves as a source of knowledge, sensory stimulation, and emotional engagement. Activities may be conducted individually or in groups and aim to promote within the community the value of the human–animal relationship as a means of achieving mutual well-being.

In Italy, the regulatory framework for AAIs is relatively stringent, as national guidelines were developed in 2015 in collaboration with the Ministry of Health [2]. Furthermore, a National Reference Center for AAIs was established to standardize operational protocols. Regarding animals involved in these interventions, the guidelines describe the required behavioural and health conditions, and state that each animal must be certified and approved by a veterinarian expert in AAIs (i.e., a veterinarian with specific training as defined in the guidelines themselves). The suitability of each animal for a specific project is assessed based on the species as well as on individual characteristics.

The guidelines emphasize strict health requirements; behavioural suitability is equally important. Animals must be free of behavioural disorders and demonstrate sociability, adaptability, the ability to spontaneously, or under guidance, interact with humans and other animals, and they must be docile. They may also undergo targeted training to develop skills for social engagement, cooperation with the handler, and motivation for activities [3,4]. Animals with recent histories of abandonment or mistreatment, including those from shelters or kennels, are generally excluded, unless they have successfully completed a rehabilitation and socialization programme supervised by a qualified behaviourist. Only adult animals are permitted, and females cannot participate during oestrus, lactation, or advanced pregnancy [3]. All health and behavioural assessments, as well as monitoring records, are carefully documented in the clinical record of each animal.

The popularity of AAIs in various settings is increasing due to their impact as an innovative tool that is able to enhance the health and well-being of different kind of users (e.g. children, elderly people, prisoners, people with disabilities).

However, due to the heterogeneity of settings [5] and to the logistics of AAIs per se, these interventions might provoke stress in the dogs involved [4]. In fact, researchers and AAI practitioners generally agree that animal welfare issues related to AAIs require special attention and official regulations. As a result, the International Association of Human–Animal Interaction Organizations IAHAIO published new guidelines to reduce work-related stress during AAIs (IAHAIO White Paper 2014, updated 2018) [6]. From a One Welfare perspective, these activities should not only improve the health of users but also safeguard the well-being of participating dogs and their handlers and, at best, be beneficial for the animals involved. Developing a multiparametric approach to monitor animal welfare and well-being during AAIs is, therefore, crucial to maintain the One Welfare concept and its prescriptions [7].

One of the best ways to prevent stress in dogs during AAIs is to choose the right and most suitable individuals for this kind of intervention. In the scientific literature, a few protocols have been developed to assess the suitability of dogs for AAIs [8].

Mongillo et al. (2015) [9] validated a two-step procedure, consisting of a behavioural examination performed by a veterinary behaviourist, followed by a role-playing simulation of an AAI session, in which dogs were exposed to unexpected social and environmental stimuli. This test represented an important step toward scientific evaluation and standardization; however, given its 10 min duration and the rapid succession of stimuli, the test may not adequately reflect the complexity and real-world conditions of actual AAI environments.

The Italian National Institute of Health proposed a set of forms and guidelines [3] for veterinarians certifying animals to be involved in AAIs. These forms are a valid method to focus on health status and broad behavioural suitability, ensuring that animals are sociable, docile, and free from behavioural disorders. However, the proposed checklists remain rather general and qualitative, offering limited detail on how to evaluate dogs’ responses to specific stimuli that may occur during interventions, and they rely heavily on the examiner’s interpretation. In fact, this scoring method may not show small differences among suitable dogs.

The Ethotest by Lucidi et al. [10] was designed to assess suitability for AAA/AAT in a three-step protocol for shelter dogs. While innovative in opening opportunities for sheltered dogs with an unknown past, it is based on a rigid scoring system. In practice, some dogs excluded by the Ethotest were later assessed as suitable according to the Delta Society (now Pet Partners) tests, highlighting the potential risks of false negatives when applying strict criteria without a margin of interpretation.

Finally, the PADA (Personality Assessment of Domestic Animals, 2023) [11] protocol proposes a highly structured protocol, including numerous practical tests (e.g., exposure to wheelchairs, crutches, screaming persons, and crowds) assessed by certified evaluators, with results stored in an international database [11]. This approach ensures transparency and standardization but requires specialized teams, resources, and certification processes, which may limit its widespread application in everyday AAI settings.

Additionally, the C-BARQ questionnaire used by Sakurama et al. [12] was proposed as a preliminary screening tool to identify promising candidates before undergoing an aptitude test. By quantifying traits such as aggression, excitability, fear, and attachment behaviours, it helps filter out unsuitable dogs early on. However, when considered alone, this approach relies entirely on caregiver reports, and thus, it does not directly measure how dogs respond to stimuli, but it does comprehend other stimuli commonly present as real-life challenges in AAI sessions.

Although existing protocols led to important advances, they still present some gaps. Current tools may not always reflect the variety of real-life AAI settings [9], rely mainly on qualitative evaluations [3], apply rigid score systems [10], depend only on subjective caregiver perception [12], or demand extensive personnel resources and certification procedures [11]. In this study, we tried to fill the procedural gaps of current AAI testing protocols by developing a novel multiparametric test, the *Suitability Test (SuiTe)* for dogs, intended to be used as a pre-screening aptitude test across all types of AAIs without distinction. By combining direct behavioural observation across a wide range of controlled but realistic stimuli, independent scoring systems, caregiver questionnaires, and physiological measures (salivary cortisol), the *SuiTe* test aims at increasing reliability, objectivity, and feasibility. Given the existence of national guidelines under a ministerial mandate, the Italian context is relatively advanced and more stringent compared to other countries. Consequently, the present data are not only relevant at the national level but could also serve as a reference point for the development of comparable frameworks in other countries.

## 2. Materials and Methods

### 2.1. Subjects

Dog–caregiver dyads were recruited by word of mouth and included both dogs already involved in AAIs and dogs exclusively kept as companions.

Dogs of all breeds and mixed-breeds, of both sexes, and regardless of neutering status, were included. Dogs could not be recruited if they were less than 1 year of age or over 14 years, in case of a current diagnosed disease (acute or chronic), as well as during ongoing pregnancy, lactation, or oestrus. Caregivers were required to be legally adults, over 18 years old, and the person who gives the most care to the dog.

The initial sample was formed by 44 dog–human dyads; three were excluded due to health issues that emerged during or immediately after the test; and another three were excluded due to the absence of a completed questionnaire by the caregiver after three reminders. The final sample size was *N* = 38.

Dogs were purebred (5 Labradors, 4 Golden Retrievers, 3 Poodles, 3 Jack Russell Terriers, 2 Maremmano-Abruzzese Sheepdog, 1 Dachshund, 1 Border Collie, 1 Breton, 1 French Bulldog, 1 Parson Russell Terrier, 1 German Shepherd working line, 1 Australian Shepherd, 1 Italian Hound, and 1 Weimaraner) and mixed-breed (12), and 39.5% of dogs were male (*N* = 15; 11 neutered and 4 intact) and 60.5% female (*N* = 23; 11 neutered and 12 intact) of different ages (mean = 6.26 ± 3.24 years; range = 1–14 years). Six of them had been previously certified as therapy dogs and participated in AAI/AAS with their caregivers. Caregivers were 6 male and 32 female of different ages (mean = 37.24 ± 16.57 years; range = 20–68 years). Of these, 25 worked with animals or had expertise in the field, while 13 did not.

### 2.2. Location and Equipment

**Figure 1:**
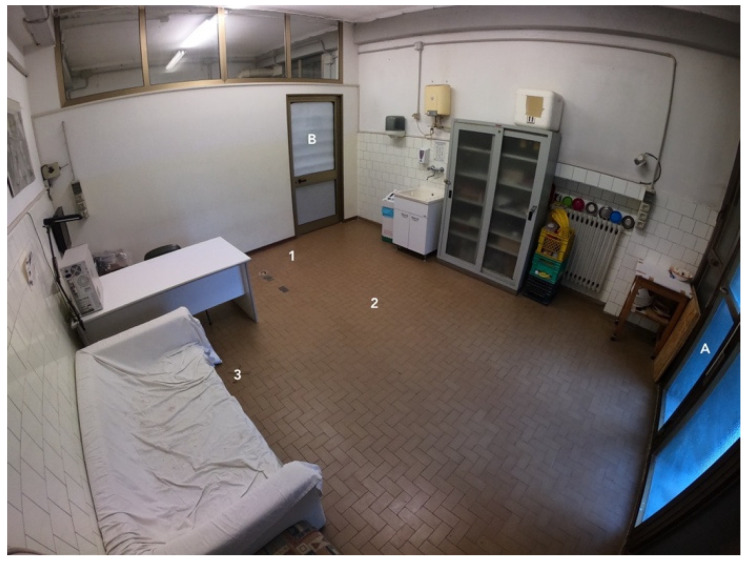
Room where the Suitability Test (*SuiTe*) was carried out (view from one of the two cameras). Doors A and B and points 1, 2, and 3 are indicated near three “X” marks, which were made using adhesive paper tape.

The tests were carried out from February to June 2025 at the Department of Veterinary Sciences—University of Pisa, Italy. The room where the test was performed measured 4 m × 5 m, with a ceramic floor where three “X” (Points 1, 2, and 3 in Figure 1) marks were made using paper adhesive tape. The three points were selected during the design of the test to facilitate its execution: Points 1 and 3 indicate where the caregiver should stand, depending on the phase being performed, while Point 2 is used to position the stimulus at the appropriate distance, as detailed in the protocol below. In addition, the points were chosen based on the position of the cameras to ensure that the dog was always visible. The room has large windows on one side and two doors (A and B), as shown in Figure 1. A desk, sofa, wardrobe, and sink were present. A box was placed on the desk, containing palatable food, toys (balls and ropes), paper sheets, and a brush; the box remained closed when not used by the operator. A paper trash bin, a metallic spoon, and two GoPro^®^ (Hero7 Black) cameras with tripods for videorecording the procedure were also required, as indicated below. Outside the room, behind door B, a wheelchair, a cleaning cart, a crutch, a white coat, a surgical face mask, sunglasses, and a baby doll were placed and kept ready for the test.

### 2.3. Experimental Protocol (SuiTe)

For this protocol, a minimum of four operators were required, as follows: (a) Operator 1 guided the protocol and remained in the room throughout the test; (b) Operator 2 assisted in forming the group and wore a white coat and surgical face mask; (c) the female stranger was a woman unfamiliar to the dog; and (d) the male stranger was a man unfamiliar to the dog.

#### 2.3.1. Preparation Phase

Before the test, caregivers were asked to bring a preferred toy and a palatable food for their dog; if they did not bring any, the researchers provided beef treats and various toys (e.g., balls, ropes). The caregiver was briefed about the protocol and asked to sign the informed consent in a room separated from where the test occurred. They were also informed that the test would be presented step-by-step by Operator 1, who would remain in the room for the entire procedure. Operator 1 explained that the dog would be gradually exposed to the test stimuli and that the protocol could be interrupted (temporarily or permanently) by leaving the room if the dog shows signs of high stress, fear, or aggression or if requested by the caregiver.

The caregiver was asked to provide information regarding the dog’s food allergies or intolerances, current health diseases or pain, and ongoing medication.

Saliva collection (T0) was then performed by the caregiver, following the instructions provided below. After this, the caregiver and the dog were accompanied to the test room and entered through entrance door A, marking the start of the procedure.

#### 2.3.2. Test Protocol

The *SuiTe* protocol was developed based on the authors’ previous experience in the field of AAIs, as well as on other behavioural tests with similar purposes, both published [9] and unpublished [3,11]. The test is divided into two main phases:

1. Environmental stimuli: Where the dog interacts with the environment, food, toys, and unusual objects to assess the dog’s motivation and behaviour in their presence;
2. Social stimuli: Where social attraction towards unfamiliar people of different genders, tolerance to handling, and reactions to “unusual” social stimuli are evaluated.

Each phase consisted of various sub-phases, described below in detail.

Phase 1: Environmental stimuli. Sub-phases:

a. Room exploration: The dyad enters through entrance door A. The dog on a leash can move around the room and the caregiver follows it. After 1 min, the leash is removed, and the dog is allowed to explore independently for another minute (total phase time: 2 min). The leash is then placed back on the dog; however, if it is too short, a choke chain, or too long, Operator 1 provides the caregiver with a leash of 1.5 m in length.
b. Food motivation: Operator 1 places a palatable food on a paper sheet on point 2. The caregiver stands on point 1 with the dog on the leash and prevents the dog from reaching the food for 10 s. After that, the dog is allowed to approach and eat the food, and the caregiver is asked to stop the dog, verbally at first; if unsuccessful in stopping the dog, they may use the leash, avoiding coercion. Finally, after removing the paper and the food, a piece of food is given to the dog from the caregiver’s open hand.
c. Toy motivation: The dog’s preferred toy (if present or chosen between a ball and a rope toy) is placed on point 2. The caregiver stands on point 1 with the dog on the leash, preventing the dog from reaching the toy for 10 s. The dog is then allowed to interact freely with the toy and play, either alone or involving the caregiver, who may invite the dog to play as they usually do. The caregiver will then ask the dog to release the toy, first verbally and, if unsuccessful, physically remove it from the dog’s mouth in a calm and non-coercive manner and without punishment. The toy is returned to Operator 1.

**Figure 2:**
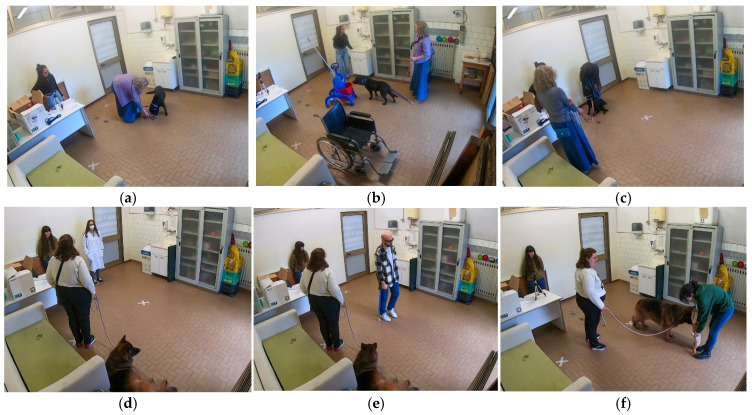
Sub-phases of the Suitability Test (*SuiTe*), respectively: (**a**) Toy motivation; (**b**) unusual static objects; (**c**) social attraction (female stranger); (**d**) white coat and surgical face mask; (**e**) crutches and sunglasses; and (**f**) baby doll.

The caregiver and the dog exit the room through door A and wait for the Operator to call them to enter. During this time, three unusual objects (a wheelchair, a cleaning cart, and a crutch) are placed in the room, as shown in Figure 2. The caregiver and the dog enter the room:

d. Unusual static objects: The caregiver and the dog, on a leash, can move around the room and explore the objects for around 10 s each.
e. Sounds: While the dog explores the objects and does not pay attention to Operator 1, a paper trash bin and then a metallic spoon are dropped to the ground. Operator 1 checks whether the dog shows signs of high stress, in which case the test is temporarily interrupted to take a break, leaving the room.
f. Unusual moving objects: While the caregiver–dog pair moves around the room, Operator 1 pushes first the cleaning cart and then the wheelchair. After a few seconds, the operator gradually approaches the dog and walks nearby.

The caregiver and the dog exit the room through door A and wait for the operator to call them to re-enter (3 min pause; the dog can drink water) after the objects are removed.

Phase 2: Response to social stimuli. Sub-phases:

g. Separation: The dog, without the leash, is free to move. The caregiver greets the dog as is usually done when leaving a familiar place (e.g., home), exits the room through door B, and leaves the leash inside. Operator 1 remains in the room with the dog, positioning himself on the wall opposite door B. Operator 1 does not initiate contact with the dog unless the dog seeks it. After 2 min, Operator 1 goes to the centre of the room, calls the dog by name, and interacts if the dog wants to and approaches (1 min).
h. Social attraction (caregiver): After 3 min outside, the caregiver enters the room through door B and stops at point 1, without interacting with or greeting the dog for 30 s. After that, the caregiver may greet the dog as usual.
i. Handling (caregiver): The dog is touched in the most sensitive areas (mouth, ears, paws, and tail), first in a gentle and then in a rougher manner; the first serves to check the potential aggressive response of the dog and the second is to simulate manipulation in AAI that might not follow common advice on how to gently handle dogs.
j. Brush (caregiver): The dog is brushed along the back for 10 s.
k. Play invitation (caregiver): Operator 1 gives the preferred toy to the caregiver to play with the dog, and then the caregiver asks the dog to release the toy verbally and, if necessary, physically.
l. Social attraction (female stranger): The dog on a leash and the caregiver stand on point 3 and wait for the entrance of the female stranger. In this phase, the dog on the leash may freely approach the stranger, and the caregiver is instructed not to prevent the dog from reaching her. The female stranger positions herself at point 1 and waits without interacting with or greeting the dog for 30 s. After that, she calls the dog by name while remaining standing, lowering her upper body, and greeting the dog by placing a hand on his head (replicating the typical and incorrect way unfamiliar people greet an unknown dog).
m. Handling (female stranger): The dog on a leash is touched in the most sensitive areas (mouth, ears, paws, and tail), first in a gentle and then in a rougher manner; the first serves to check the potential aggressive response of the dog and the second is to simulate manipulation in AAI that might not follow common advice on how to gently handle dogs.
n. Brush (female stranger): Operator 1 gives the brush to the stranger, and the dog is brushed along the back for about 10 s.
o. Play invitation (female stranger): Operator 1 gives the preferred toy to the female stranger to play with the dog, and then the dog is asked to release the toy verbally and, if necessary, physically. After that, the female stranger leaves the room.
p. Social attraction (male stranger): The dog is on a leash, and the caregiver stands on point 3 and waits for the entrance of the male stranger. In this phase, the dog on the leash may freely approach the stranger, and the caregiver is instructed not to prevent the dog from reaching him. The male stranger positions himself on point 1 and waits without interacting with or greeting the dog for 30 s. After that, he calls the dog by name while remaining standing, lowering his upper body, and greeting the dog by placing a hand on its head (replicating the typical and incorrect way unfamiliar people greet an unknown dog).
q. Handling (male stranger): The dog on a leash is touched in its most sensitive areas (mouth, ears, paws, and tail), first in a gentle and then in a rougher manner; the first serves to check the potential aggressive response of the dog and the second is to simulate manipulation in AAI that might not follow common advice on how to gently handle dogs.
r. Brush (male stranger): Operator 1 gives the brush to the stranger, and the dog is brushed along the back for about 10 s.
s. Play invitation (male stranger): Operator 1 gives the preferred toy to the male stranger to play with the dog, and then the dog is asked to release the toy verbally and, if necessary, physically.
t. Social attraction (group): The female stranger and Operator 2 enter the room and place themselves near the male stranger, forming a group. The group remains quiet for 10–15 s while standing, then talks to each other with a normal tone of voice for 10–15 s, and, finally, talks to each other in a loud voice for 10–15 s. In this phase, the dog on the leash and the caregiver stand on point 3 and may freely approach the group; the caregiver is instructed not to prevent the dog from reaching them.
u. Handling (group): The group calls the dog by name. In this phase, the dog on the leash may freely approach the group, and the caregiver is instructed not to prevent the dog from reaching them if the dog wants to. If the dog approaches, the group greets and pets the dog simultaneously. The group exits the room (door B).
v. White coat and surgical face mask: Operator 2 enters the room (door B) wearing a white coat and a surgical face mask, stands on point 1, and does not interact with the dog for 30 s. The operator then leaves the room (door B).
w. Crutches and sunglasses: The male stranger enters the room (door B) and walks with a limp, moving around and passing near the dog twice. He then leaves the room (door B).
x. Baby doll: The female stranger enters the room (door B) holding a baby doll in her arms. After a few seconds, she puts it down and pretends to make it walk by holding it by the arms, as if it were a child [13]. She then leaves the room (door B).
y. Training and concentration: The caregiver asks the dog to perform some commands (e.g., sit, stay, paw, or others the dog knows). In the middle of the exercise, an operator from outside the room knocks on door B and briefly appears, says a phrase, and then closes the door. The caregiver tries to keep the dog focused on the exercise.
z. Sofa: The caregiver asks the dog to go up the sofa.

After the test, the dog and the caregiver remain in the room to immediately collect the second saliva sample (T1). The entire test protocol takes approximately 45 min.

#### 2.3.3. Assessment of the Dog Behavioural Responses

Two independent evaluators observed the video-recorded tests using ad hoc scoring grids and a classification. The *SuiTe* protocol and relative scoring grids were developed by adapting and integrating items from a validated test protocol [9], as well as from protocols not yet validated [3,10,11], and from the prior experience of the authors in the field of AAIs and behavioural testing.

The *SuiTe* evaluation consisted of a mixed objective–subjective approach, based on X-Y scores derived from “valence” and “arousal” dimensions of the circumplex model of affect [14] and on the descriptions provided in the four-quadrant Cartesian grid. The score attribution was performed using two primary dimensions, valence and arousal. These dimensions provide a framework for organizing and interpreting emotional responses [15]:

- Valence: Refers to the positive or negative character of an emotion [16]. Positive valence refers to approach, retaining, tolerance, and acquisition; negative valence refers to withdrawal, escape, refusal, and aggression [16]. In other words, it reflects whether an experience was perceived as unpleasant, e.g., fear or frustration, or pleasant, e.g., contentment or play [17].
- Arousal: Represents the activation level of an emotional response, independent of its valence. High levels of arousal can be manifested as excitement or agitation, while low arousal corresponds to calm or passive states [18].

The evaluators’ scoring grid was structured as a Cartesian plane, with the x-axis representing Valence (−5 ≤ x ≤ +5; positive x > 0 and negative x < 0) and the y-axis representing Arousal (−5 ≤ y ≤+5; y > 0 for high levels and y < 0 for low levels) of the behaviours observed during the test. For sub-phase a-x, the evaluators assigned a coordinate (x; y) based on the dog’s behaviour, following a training period with these grids. To facilitate evaluation, a description of each quadrant was provided, illustrating the extreme behavioural expressions at the coordinates (−5,−5, e.g., the dog does not want to interact with people), (−5, +5, e.g., the dog tries to escape the room and actively avoids interaction), (+5, +5, e.g., intense attempts to seek physical contact with a stranger, such as jumping), and (+5, −5, e.g., the dog remains calm, does not actively seek physical contact, and shows interest in the environment). A “Notes” section was also provided to the evaluators to record observed behaviours, including aggressiveness when it occurred. Aggressiveness was scored as −5 for valence and +5 for arousal. The same valence score was also assigned when the dog refused to participate in or complete a sub-phase (e.g., remaining distant from unfamiliar people and avoiding interaction or physical contact). For sub-phases y–z, an independent score was assigned using another grid, which will be discussed in a future investigation.

After an overall evaluation, each dog was classified as suitable (S), unsuitable (U), or pending suitability (P), similar to a classification used in a previously validated test for the suitability of dogs in AAIs [9]. Translating the characteristics of a “suitable dog” into scores was not simple; the evaluation needed to recognize as suitable both slightly excitable and active dogs, as well as calmer and more relaxed ones, recognizing that these two contrasting profiles are not mutually exclusive in terms of suitability. Furthermore, the capacity for recovery is one of the aspects contributing to dog suitability and influencing the score assigned to each phase. For instance, during the scoring of the “Sounds” phase, a dog that was initially startled but quickly recovered and approached the stimulus received a score indicating a more positive valence, compared to a dog that remained unsettled after the noise and did not return to a calm state.

**Figure 3:**
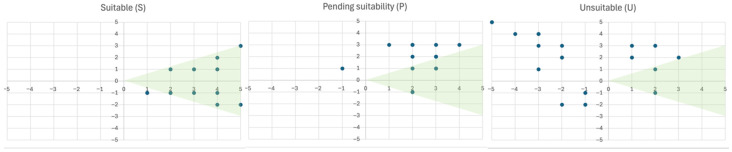
These grids represent an example of, respectively, the following: suitable, pending suitability, and unsuitable subjects. Every point on the Cartesian grid is an X-Y score given by the evaluators as valence–arousal (−5 ≤ x; y ≤ +5). A triangular green area indicates the suitability area. The S and P images have fewer points on the Cartesian grid than the U, because when two scores are repeated more than once, the two points will overlap.

In the *SuiTe*, a “suitable dog” was defined as one displaying behaviours with a positive valence and an arousal level fluctuating between positive and negative values, gradually increasing as valence shifted toward more positive levels. This pattern formed a triangular area with the vertex at 0,0 and the other two vertices approximately at +5 for valence and +3/−3 for arousal, as illustrated in Figure 3. Dogs classified as suitable (S) were expected to obtain the majority of their scores within this triangular green area (Figure 3), with particular attention given to performance in the most relevant sub-phases (e.g., exposure to unusual objects such as wheelchairs, responses to sounds, or handling by unfamiliar people). The triangular area represents dogs ranging from proactive in social interaction and slightly excitable to calm and relaxed when approached, without being easily excitable. If a dog obtained all scores within the triangular area except in the food-motivation sub-phase, the dog could still be classified as suitable (S), provided that overall valence remained positive. Conversely, if a dog obtained most scores within the area but exhibited high arousal in certain sub-phases (e.g., intense attempts to seek physical contact with a stranger, such as jumping), the dog could be classified as P, with this aspect highlighted to the caregiver so that the dyad could work on it. Finally, if a dog obtained the majority of scores outside the triangular area and with negative valence, the dog was classified as unsuitable (U). The evaluators were responsible for assessing each case individually and considering the overall behaviour of the dog–caregiver dyad throughout the entire test.

### 2.4. Questionnaire

In addition, caregivers were asked to complete an online, purposefully developed questionnaire incorporating items from the validated Italian version of the C-BARQ [19], as previously performed by [12,20], as well as newly introduced items designed to assess dogs’ behaviour in response to the stimuli administered during the behavioural test. A copy of the questionnaire is provided in the Supplementary Materials.

### 2.5. Saliva Sample Collection and Cortisol Analysis

Saliva samples were collected from the dog using Salivette^®^ (Sarstedt, Rommelsdorft, Germany) swabs, which were chewed for approximately 90 s between the molars, both before the test (T0) and immediately after its end (T1). The saliva samples were refrigerated at −20 °C and stored at this temperature in the laboratory of the Department of Veterinary Sciences (University of Pisa, Italy) until analysis. Cortisol quantification was performed using the Salimetrics Cortisol Enzyme Immunoassay Kit^®^ (Salimetrics, Segrate, Italy), as reported in a previous study [21].

### 2.6. Statistical Analysis

Descriptive statistics were performed, and data are presented as median (with interquartile range [IQR] and minimum–maximum values), unless otherwise stated. Data were analyzed using RStudio software (Version 2024.12.0+467).

*SuiTe*: The inter-rater reliability between two independent evaluators was calculated using the Intraclass Correlation Coefficient (ICC). A non-parametric Kruskal–Wallis test (*p* < 0.05), followed by a post hoc Dunn test with Holm correction, was performed to assess potential differences in valence and arousal median scores between the three groups (suitable, unsuitable, or pending suitability). To further investigate these aspects and groups’ differences, and to increase group sizes for more appropriate statistical analysis (*N* = 14 vs. 9 and 5), the suitable and pending suitability groups were merged, and a non-parametric Mann–Whitney U test (*p* < 0.05) was performed (suitable and pending suitability vs. unsuitable).

Salivary cortisol: Normality was assessed using the Shapiro–Wilk test, which revealed that salivary cortisol values were not normally distributed (T0: W = 0.68568, *p* = 2.249 × 10^−7^; T1: W = 0.80792, *p* = 2.928 × 10^−5^). Because the data were not normally distributed, a non-parametric Wilcoxon paired signed-rank test (*p* < 0.05) was performed to examine potential differences in cortisol levels at T0 and T1 between the three (suitable, unsuitable, and pending suitability) and also between the two groups (suitable and pending suitability vs. unsuitable). Delta cortisol levels (T1−T0) were calculated, and a non-parametric Kruskal–Wallis test (*p* < 0.05) was conducted to assess potential differences in delta cortisol levels between the three groups and a non-parametric Wilcoxon signed-rank sum test (*p* < 0.05) between the two groups.

Questionnaire scores: A non-parametric Kruskal–Wallis test (*p* < 0.05), followed by a post hoc Dunn test with Holm correction, was conducted to assess potential differences in mean scores between the three (suitable, unsuitable, and pending suitability) and also between the two groups (suitable and pending suitability vs. unsuitable), considering the different sections of the questionnaire (trainability and obedience, separation, aggression, fear and anxiety, excitability, attachment behaviours, and other behaviours).

In addition, the open-ended responses provided by the caregivers to the final question of the Italian questionnaire were considered in the analysis:

*“In your opinion, would your dog be suitable for participating in Animal-Assisted Interventions (also known as pet therapy)? Please answer Yes/No and explain the reason for your response”.* The open-ended responses provided by the caregivers, reported in the Results section, were simplified and classified by a researcher into four categories: “yes”, “yes after training/course”, “maybe”, and “no.” A qualitative analysis was performed to evaluate the level of concordance between the caregivers’ perception of suitability and the results of the *SuiTe* test, and Cohen’s kappa was calculated.

## 3. Results

### 3.1. SuiTe

After an overall evaluation of the 38 subjects, 24 dogs were classified as unsuitable (U), 9 as suitable (S), and 5 as pending suitability (P) according to the test. For arousal, inter-rater reliability was moderate (ICC = 0.605, 95% CI: 0.563–0.645, *p* < 0.001), and for valence, inter-rater reliability was good (ICC = 0.699, 95% CI: 0.665–0.731, *p* < 0.001). Descriptive statistics for the test’s scores are reported in Table 1.

**Figure 4:**
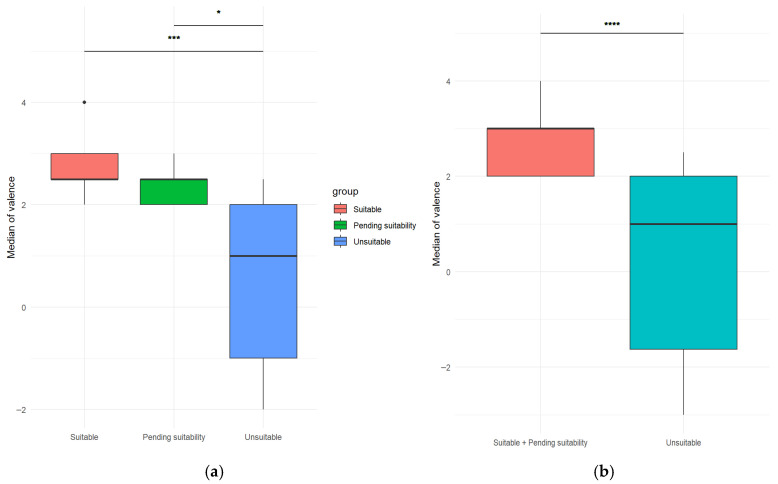
Significant difference in median of valence, according to the following: (**a**) Kruskal–Wallis test (*p* < 0.05) across groups (suitable, pending suitability, unsuitable, respectively red, green and blue). Significance codes: ‘***’ < 0.001; ‘*’ < 0.05; (**b**) Mann–Whitney U test (*p* < 0.05) across two groups (suitable + pending suitability vs. unsuitable, respectively red and light blue). Significance codes: ‘****’ < 0.0001.

A Kruskal–Wallis test (*p* < 0.05) followed by a post hoc Dunn test with Holm correction revealed a statistically significant difference in valence scores across groups (suitable, pending suitability, unsuitable; χ^2^(2) = 19.5, *p* < 0.0001) with a large effect size (η^2^ = 0.449) (Table 2 and Figure 4a), whereas no significant difference was observed in arousal scores across groups (χ^2^(2) = 3.89, *p* > 0.05).

To further investigate groups’ differences, the suitable and pending suitability groups were merged. A Mann–Whitney U test revealed that the median valence was significantly lower in the unsuitable group compared with the merged suitable/pending group (U = 25.5, *p* < 0.0001), with a large effect size (r = 0.71) (Figure 4b).

For median arousal, the Mann–Whitney U test showed no significant difference between the unsuitable and suitable/pending groups (U = 226, *p* = 0.078), with a small effect size (r = 0.29).

### 3.2. Questionnaire

A Kruskal–Wallis test (*p* < 0.05) was performed on the mean scores of each section of the questionnaire (trainability and obedience, separation, aggression, fear and anxiety, excitability, attachment behaviours, and other behaviours) for each subject, comparing the three groups (suitable, pending suitability, and unsuitable), as reported in Table 3. For each section, the type of scores is also reported, as the questionnaire requested indicating either the frequency or the intensity of a behaviour according to the corresponding scale (Supplementary Materials).

Post hoc Dunn test with Holm correction was performed for the section that had a *p*-value < 0.05 or with a significant tendency in the Kruskal–Wallis test, as reported in Table 4.

Dogs classified in the test as suitable and pending suitability showed lower scores for separation (S + P: mean ± standard deviation 0.205 ± 0.223 vs. U: 0.677 ± 0.613), attachment (S + P: 1.857 ± 0.727 vs. U: 2.424 ± 0.815), and fear/anxiety (S + P: 0.493 ± 0.379 vs. U: 0.903 ± 0.520) in the questionnaire. Considering aggression, U showed higher mean scores than S and P (U: 16.750 ± 11.674 vs. S + P: 8.857 ± 6.815).

To further investigate groups’ differences, the suitable and pending suitability groups were merged and a Mann–Whitney U test (*p* < 0.05) revealed that the scores of each section of the questionnaire (trainability and obedience, separation, aggression, fear and anxiety, excitability, attachment behaviours, and other behaviours) for each subject, comparing the two groups (suitable and pending suitability vs. unsuitable), as reported in Table 5.

### 3.3. Cortisol Levels

Before the analysis, three dogs (six samples: T0 and T1 for each) were excluded due to insufficient saliva in the swabs. The final sample size for salivary cortisol measurement was 70 (T0 and T1, Table 6). Dogs classified in the test as suitable, pending suitability, or unsuitable showed the following mean (± standard deviation) of cortisol concentration at T0 and T1, respectively: suitable (T0: 2.36 ± 2.32; T1: 2.22 ± 1.56), pending (T0: 2.21 ± 1.22; T1: 1.70 ± 0.33), and unsuitable (T0: 1.15 ± 0.74; T1: 1.36 ± 0.73).

The Wilcoxon signed-rank test revealed no statistically significant differences between T0 and T1 either within each of the three groups (Suitable: V = 16, *p*-value = 0.8438; pending suitability: V = 11, *p*-value = 0.4375; unsuitable: V = 82, *p*-value = 0.156) nor within the two groups (suitable + pending suitability: V = 53, *p*-value = 0.6355; unsuitable: V = 82, *p*-value = 0.156). Delta cortisol values did not reveal statistically significant differences neither across the three groups (Kruskal–Wallis test, χ^2^ (2) = 1.73, *p* = 0.42) nor between S + P versus U dogs (Wilcoxon signed-rank sum test, W = 112, *p* = 0.3023, 95% C.I. −1.1080636; 0.2216536; r = −0.321).

### 3.4. Questionnaire Qualitative Analysis

All open-ended responses provided by the caregivers to the final question of the survey were simplified by summarizing the main characteristics (e.g., tolerant, sociable, playful, and excitable) indicated by the caregivers based on their perception of the dog’s presumed behaviour and classified into four categories (“Yes,” “Yes after a training program,” “Maybe,” and “No”), as reported in Table 7, and “Yes” corresponds to suitable, “No” to unsuitable, and “Maybe” and “Yes after training” to pending suitability.

Considering the questionnaire responses, 22 dogs were considered as suitable (vs. 9 according to the test), 11 as unsuitable (vs. 24 according to the test), and 5 as pending suitability (vs. 5 according to the test but not matching). Cohen’s kappa indicated a fair agreement of 0.285. The concordance is better illustrated in Table 8.

## 4. Discussion

This preliminary study aimed to assess the potential of two combined tools, a behavioural aptitude test (Suitability Test *SuiTe*) and a survey (an ad hoc revised questionnaire incorporating C-BARQ), to be used as pre-screening for dog suitability in AAIs/AAS. In addition, salivary cortisol levels were measured to include a physiological indicator of the dogs’ stress response to the stimuli presented during the test. Results showed that differences exist between dogs classified as suitable (S) or pending suitability (P) versus unsuitable (U), both in the valence scores obtained in *SuiTe* and in separation, attachment, and fear/anxiety behaviours as assessed by the questionnaire; and these differences were aligned. However, suitability as assessed by the *SuiTe* resulted in lower values compared to suitability assessed by caregivers through an open question.

Dogs judged suitable obtained scores with more positive values compared to unsuitable ones. In fact, when considering the mean valence scores across all sub-phases together, values were >2 for S and P dogs, whereas they were close to zero for U dogs. Therefore, the test could overall be considered not excessively stressful or challenging. On the other hand, if the SuiTe—designed as a pre-screening for suitability—were too demanding for the dogs in terms of emotional state, more dogs (especially U) would likely have exhibited negative valence during the test. Although it would have been useful to analyze the responses to each stimulus separately within the groups, in order to determine which of them had the greatest impact, this will be investigated in the future with a larger sample. For this pilot study, a different approach was chosen considering that, in a real AAI session, stimuli can occur altogether, at the same time, or in a limited time, and thus the overall valence is relevant. For instance, an unsuitable dog that is scared by a sudden noise may subsequently stop interacting with people, given the possible presence of a carryover effect. One of the strengths of suitable dogs should be the ability to manage even situations that can lead to mild discomfort, particularly with the support of their caregiver. Another strength of suitable dogs is that they can quickly recover in case something unpredictable and unpleasant occurs, as this sometimes may happen in AAI sessions.

The results of this study, in fact, suggest that suitable dogs have characteristics like interspecific sociability and tolerance, curiosity toward stimuli, and willingness to approach without fear, as well as focus and engagement with stimuli around them. As for arousal levels, suitable dogs varied, shifting towards more central ranges, but no statistically significant differences were found between S, P, and U dogs, either in their test behaviours or in the questionnaire scores (excitability scale). It may reflect the diversity among dogs considered suitable in the test (ranging from slightly positive and slightly negative arousal), from calm and relaxed individuals to more lively and energetic ones, matching the type of people they are expected to work with and the type of activities (individual or group). Indeed, *SuiTe* aimed to detect dogs with the appropriate aptitude to participate in AAIs in a preliminary phase of selection, and including dogs that exhibit moderate variations in arousal levels seems appropriate. Companion dogs do not request to participate in AAIs, nor do they voluntarily consent to be involved. Assessing their emotional state during these activities—ensuring that they do not experience stress and, ideally, that they enjoy the interaction [22]—is a fundamental aspect of both the selection and the ongoing re-evaluation process, representing an essential responsibility on our part [23,24].

Cortisol concentrations in saliva were within the physiological range [25,26], with mean values higher than those reported in a previous study conducted under similar conditions and in the same laboratory [21]. The analysis did not reveal differences based on suitability; it can be hypothesized that, when negative valence occurred, the dog’s physiological response was not strong enough to elicit marked cortisol peaks, even in unsuitable dogs. The observed differences between the reported raw *p*-values and the Holm-adjusted *p*-values (Table 4) may result from the multiple pairwise comparisons among the three groups and the application of the Holm correction, which increases *p*-values, particularly those close to the significance threshold. In addition, the lack of a significant difference may suggest that the test is valid from an animal welfare perspective, as it does not elicit strong stress responses [27]. At the same time, however, since it does not elicit strong stress, it may have limited effectiveness for assessment, as it does not provide additional information beyond the behavioural response of the dog, which is indeed what is strictly necessary for assessing suitability in AAIs. Data obtained from a bigger sample should confirm these results or give a more nuanced picture.

Dogs classified in the test as suitable and pending suitability showed lower mean scores of separation, attachment, and fear/anxiety sections, as similarly reported by Sakurama et al. [12], except for the attachment. Separation and attachment sections were linked to *SuiTe* sub-phase g, as the separation response, which typically is a stressful moment for most dogs and may be influenced by the quality of the attachment bond established within the dyad [21]. Moreover, the tendency in the attachment section may suggest different attachment behaviours between S and U. The fear/anxiety section is expressed throughout the test (in response to both environmental and social stimuli), and whenever fear was observed, these corresponded to negative valence scores. Thus, the confirmation of the existing difference in caregivers’ perception of behaviours between S and U may indicate that dogs in S (and S + P) exhibit less fear/anxiety than those in the U group, as well as fewer problems with separation from the caregiver, as previously reported in the literature [12]. The next step of this project will consist of better assessing the relevance of the bond between dogs and their caregivers [28], which might give new insights for the interpretations of these findings.

Interestingly, when analyzing S, P, and U separately, the aggressiveness section of the questionnaire did not reveal any significant differences; however, when S and P were combined, a significant difference emerged (higher in U vs. S + P). Indeed, the presence of aggressiveness is one of the most important factors leading to the classification of a dog as unsuitable [9,12]. Ensuring proper selection, training, and management of dogs in AAIs is essential not only for safeguarding their welfare but also for preventing undesirable scenarios, including aggression and potential bites, during sessions [29].

Taking into consideration the results of both the tests and the questionnaires, it appears that a moderate agreement exists. These findings are consistent with previous studies, in which the C-BARQ was considered a useful tool to help caregivers evaluate whether their dogs had a temperament making them suitable for becoming therapy dogs [12]. However, the agreement decreased for the overall suitability. In fact, in this study, we also asked caregivers to say whether and why their dogs were, in their opinion, suitable for participating in animal-assisted interventions; it emerged that the overall concordance with *SuiTe* was fair. This means that only half of the caregivers provided an accurate assessment, according to our *SuiTe* evaluations. When caregivers considered the dogs unsuitable, *SuiTe* provided the same evaluation in almost all cases. Conversely, only 41% of those who believed their dogs were suitable were correct based on *SuiTe* evaluations. A possible explanation is that caregivers may have a subjective perception of their dogs and, wishing they are suitable, unconsciously overestimate their abilities, not knowing what AAIs consist of, and therefore what their dogs would be exposed to. This aspect may suggest that, in accordance with Mongillo et al. [9], behavioural evaluation through testing represents the better assessment for identifying suitability for AAIs. Moreover, results indicated that caregivers may overestimate desirable behaviours for AAIs in their dogs or underestimate behaviours that render dogs unsuitable for AAIs (e.g., the dog does not want to be pet by a stranger, does not approach a group of unfamiliar people when called, shows fear or anxiety in the presence of unusual objects, or becomes stressed by unfamiliar people [9,12]), but more investigations are needed. These aspects can be particularly concerning when considering the everyday life of the dyad and the welfare of the dog. Caregivers may not always be able to detect stressful situations for their animals or recognize certain stress signals exhibited by the dog [30]; this can, in turn, lead caregivers to force the dog to cope with difficulties, possibly without the caregiver’s support [31,32]. From this perspective, *SuiTe* could also represent a valuable tool for dogs that will never participate in AAIs, as it may help identify potential difficulties in their everyday life.

*SuiTe* presents some innovation compared to previous tests. The overall duration of the test was extended to approximately 45 min, and a defined time to each sub-phase was established in order to improve standardization and repeatability. Differently from the test published by [9], which includes a role-playing component that is probably closer to real-life situations because different stimuli are presented together, and in *SuiTe* the environmental and social stimuli were separated into two distinct phases and subsequently into sub-phases. This approach allows for a more precise identification of the type of stimuli to which the dog’s response is related; in the future, an evaluation assigning a specific score to each sub-phase might provide valuable information, although the current study showed that dogs were assessed as unsuitable when the overall valence was low, suggesting that the negative score for one stimulus was usually associated with negative scores to other stimuli. Moreover, this tool, thanks to its graphical output, allows researchers and caregivers to clearly identify which *SuiTe* stimuli are most challenging for the dog.

The ultimate aim of *SuiTe* is not to determine whether a dog is currently ready to participate in AAIs, but rather to assess his aptitude as a screening test before starting a training programme. In fact, if a caregiver wishes to begin an educational programme to become a handler in AAIs and to certify the dog, *SuiTe* can help identify the specific aspects that require further work.

Future analyses will attempt to address some limitations of this study. The sample size will be expanded by including new dyads, and a more detailed and deeper evaluation will be conducted on the comparison between each sub-phase of the *SuiTe* and the scores of specific correlated questions. Such analyses may provide a more accurate assessment of the efficiency of individual sub-phases in reflecting what is reported by caregivers. Moreover, some stimuli are missing in *SuiTe*, for ethical reasons, such as interactions with children and older adults, as well as other additional situations like an off-leash moment with strangers, food being offered by a stranger, and interactions with people in a wheelchair or lying in bed, which could be added to further refine the protocol. However, the *SuiTe* should maintain agility and feasibility; therefore, this represents a compromise, and integration with the survey is necessary to cover some unexplored aspects. In addition, a possible follow-up after one year or more, repeating the test, could provide important information on whether and how these responses change over time, particularly in young dogs. Finally, a better evaluation of the dog–caregiver bond might provide relevant inputs.

## 5. Conclusions

The evaluation of dog suitability for their involvement in AAIs is complex, due to the vast variety of characteristics required and the settings in which AAIs are performed. *SuiTe* can serve as a useful screening tool, both for caregivers who wish to begin the training for animal-assisted interventions and for experienced handlers, e.g., in case they are considering retiring their dogs or to evaluate the suitability of a new one. Furthermore, *SuiTe* may provide preliminary information to support veterinarians specialized in AAIs, who, in Italy, hold the responsibility for certification, as well as other experts in this field. Integrating its results with questionnaire data and hormonal assessments could offer additional insights, contributing to a more comprehensive overall evaluation of the dyad.

## Figures and Tables

**Table 1:** Descriptive statistics of the test’s scores with variable (valence, arousal), group (suitable, pending suitability, and unsuitable), number of scores (N), mean, median, IQR (Q1–Q3), minimum, and maximum.

Variable	Group	N (Scores)	Mean	Median	Q1	Q3	Minimum	Maximum
Valence	Suitable	216	2.5	2.5	2	3	−1.5	5
	Pending suitability	119	2.1	2.5	1.5	3	−2	4.5
	Unsuitable	577	0.4	1	−1.5	3	−5	4
	All	912		2	0	3	−5	5
Arousal	Suitable	216	1.0	1.5	0.25	2	−2	4
	Pending suitability	119	1.2	1.5	0	3	−3	5
	Unsuitable	577	1.5	2	0.5	3	−5	5
	All	912		1.5	0.4	2.5	−5	5

**Table 2:** Post hoc Dunn test with Holm correction for valence scores with a comparison between the three groups (suitable, pending suitability, unsuitable); the test statistic, adjusted *p*-values (Holm correction), and significance codes are reported. Significance codes: ‘***’ < 0.001; ‘*’ < 0.05; ‘ns’ = not significant.

Comparison	Test Statistic	*p*-Value	Adjusted *p*-Value	Significance Codes
Suitable vs. Unsuitable	−4.11	<0.0001	<0.001	***
Suitable vs. Pending suitability	−0.704	0.481	0.481	ns
Pending suitability vs. Unsuitable	−2.47	0.014	0.0273	*

**Table 3:** Kruskal–Wallis test (*p* < 0.05) for mean scores of each questionnaire section (trainability and obedience, separation, aggression, fear and anxiety, excitability, attachment behaviours, and other behaviours), indicating the type of scores (frequency or intensity), number of variables (n), test statistic, df, *p*-values with significant codes, effect size (η^2^), and relative magnitude (small, moderate, or large). Significance codes: (^.^) = significant tendency; and (*) = < 0.05.

Questionnaire Section	Type of Score	n	Test Statistic	df	*p*-Value	Effect Size (η^2^)	Magnitude
Trainability and obedience	Frequency	38	1.35	2	0.509	−0.0186	small
Separation	Frequency	38	8.06	2	0.018 (*)	0.173	large
Aggression	Intensity	38	5.03	2	0.081	0.0865	moderate
Fear and anxiety	Intensity	38	6.34	2	0.042 (*)	0.124	moderate
Excitability	Intensity	38	1.13	2	0.568	−0.0249	small
Attachment behaviours	Frequency	38	5.41	2	0.067 (^.^)	0.0974	moderate
Other behaviours	Frequency	38	3.41	2	0.182	0.0403	moderate

**Table 4:** Post hoc Dunn test with Holm correction, comparing suitable vs. unsuitable; suitable vs. pending suitability; unsuitable vs. pending suitability for each questionnaire section that had a *p*-value < 0.05 or with a significant tendency at the Kruskal–Wallis test. Test statistic, *p*-value, and adjusted *p*-value are reported. Significance codes: (^.^) = significant tendency.

Questionnaire Section	Comparison	Test Statistic	*p*-Value	Adjusted *p*-Value
Separation	Suitable vs. Unsuitable	2.39	0.017	0.051 (^.^)
	Suitable vs. Pending suitability	−0.099	0.920	0.920
	Unsuitable vs. Pending suitability	−2.01	0.044	0.088 (^.^)
Fear and anxiety	Suitable vs. Unsuitable	1.93	0.054	0.136
	Suitable vs. Pending suitability	−0.413	0.68	0.68
	Unsuitable vs. Pending suitability	−2.00	0.046	0.136
Attachment behaviours	Suitable vs. Unsuitable	2.33	0.020	0.060 (^.^)
	Suitable vs. Pending suitability	1.15	0.251	0.502
	Unsuitable vs. Pending suitability	−0.547	0.585	0.585

**Table 5:** Mann–Whitney U test (*p* < 0.05) for mean scores of each questionnaire section (trainability and obedience, separation, aggression, fear and anxiety, excitability, attachment behaviours, and other behaviours), indicating the type of scores (frequency or intensity), number of variables (n1 = unsuitable; n2 = suitable + pending suitability), test statistic, df, *p*-values with significant codes, effect size (η^2^), and relative magnitude (small, moderate, or large). Significance codes: (*) = < 0.05; and (**) = < 0.01.

Questionnaire Section	Type of Score	n1 vs. n2	Test Statistic	*p*-Value	Effect Size (η^2^)	Magnitude
Trainability and obedience	Frequency	24 vs. 14	133	0.292	0.173	Small
Separation	Frequency	24 vs. 14	260	0.0048 (**)	0.460	moderate
Aggression	Intensity	24 vs. 14	242	0.027 (*)	0.361	moderate
Fear and anxiety	Intensity	24 vs. 14	250	0.0136 (*)	0.403	moderate
Excitability	Intensity	24 vs. 14	198	0.363	0.150	small
Attachment behaviours	Frequency	24 vs. 14	234	0.0447 (*)	0.328	moderate
Other behaviours	Frequency	24 vs. 14	184	0.65	0.076	small

**Table 6:** Descriptive statistics of salivary cortisol concentrations are reported for each variable (cortisol concentration at T0 and T1, delta cortisol levels, and overall cortisol levels), including the number of samples (N), mean, standard deviation (SD), median, interquartile range (Q1–Q3), minimum, maximum, and results of the Shapiro–Wilk test (W and *p*-value; *p* < 0.05 indicates non-normal distribution).

Variable	N	Mean	SD	Median	Q1	Q3	Min	Max	W	*p*-Value
Cortisol concentration ng/mL (T0)	35	1.580	1396	1.204	0.812	1.539	0.231	7.314	0.68568	<0.001
Cortisol concentration ng/mL (T1)	35	1.607	0988	1.379	1.093	2.071	0.143	5.855	0.80792	<0.001
Δ cortisol (T1−T0)	35	0.027	0820	0.138	−0.232	0.459	−1.952	1.329	0.93169	0.0314
All cortisol levels (T0 + T1)	70	1.594	1200	1.268	1.033	1.788	0.143	7.314	0.74426	<0.001

**Table 7:** Qualitative analysis of open-ended responses for each subject after simplification by the researchers, with corresponding categorization (categories: “Yes,” “Yes after a training program,” “Maybe,” and “No”), expected classification at the test considering the caregiver’s open-ended responses, and the real classification according to the test. Concordance or discordance is indicated, considering that Yes = suitable; No = unsuitable; and Maybe and Yes after a training programme = pending suitability.

Code	Simplified Open-Ended Responses	Categories	Expected Classification at the Test Based on Caregiver’s Response	Classification Emerged by the Behavioural Test (*SuiTe*)	Agreement/Disagreement
1	Yes, tolerant	Yes	Suitable	Unsuitable	Disagreement
2	No, excitable dogs; stress with strangers; not interested in play	No	Unsuitable	Unsuitable	Agreement
3	Yes	Yes	Suitable	Suitable	Agreement
4	No, fearful	No	Unsuitable	Unsuitable	Agreement
5	No, stressed	No	Unsuitable	Unsuitable	Agreement
6	Yes	Yes	Suitable	Unsuitable	Disagreement
7	No	No	Unsuitable	Unsuitable	Agreement
8	Yes, cuddly	Yes	Suitable	Unsuitable	Disagreement
9	No	No	Unsuitable	Unsuitable	Agreement
10	I don’t know	Maybe	Pending suitability	Unsuitable	Disagreement
11	No, not interested in play and stranger	No	Unsuitable	Unsuitable	Agreement
12	No, excitable	No	Unsuitable	Pending suitability	Disagreement
13	Yes, after a training programme	Yes after a training programme	Pending suitability	Unsuitable	Disagreement
14	No, excitable	No	Unsuitable	Unsuitable	Agreement
15	Yes, after a training programme	Yes after a training programme	Pending suitability	Pending suitability	Agreement
16	I don’t know	Maybe	Pending suitability	Unsuitable	Disagreement
17	Yes	Yes	Suitable	Suitable	Agreement
18	Yes	Yes	Suitable	Suitable	Agreement
19	No, fearful and stressed	No	Unsuitable	Unsuitable	Agreement
20	Yes, sociable	Yes	Suitable	Unsuitable	Disagreement
21	Yes, sociable and balanced	Yes	Suitable	Suitable	Agreement
22	Yes, sociable and tolerant	Yes	Suitable	Suitable	Agreement
23	Yes, sociable and obedient	Yes	Suitable	Unsuitable	Disagreement
24	Yes, playful and obedient	Yes	Suitable	Suitable	Agreement
25	Yes, calm and sociable	Yes	Suitable	Unsuitable	Disagreement
26	Yes, sociable	Yes	Suitable	Unsuitable	Disagreement
27	Yes tolerant and playful	Yes	Suitable	Unsuitable	Disagreement
28	Yes	Yes	Suitable	Suitable	Agreement
29	Yes, encourages affection	Yes	Suitable	Suitable	Agreement
30	Yes sociable and playful	Yes	Suitable	Unsuitable	Disagreement
31	No, stressed	No	Unsuitable	Unsuitable	Agreement
32	I don’t know	Maybe	Pending suitability	Unsuitable	Disagreement
33	No, not sociable	No	Unsuitable	Unsuitable	Agreement
34	Yes, tolerant	Yes	Suitable	Pending suitability	Disagreement
35	Yes, cuddly	Yes	Suitable	Unsuitable	Disagreement
36	Yes	Yes	Suitable	Suitable	Agreement
37	Yes, tolerant	Yes	Suitable	Pending suitability	Disagreement
38	Yes	Yes	Suitable	Pending suitability	Disagreement

**Table 8:** Contingency table between caregivers’ responses (column) and *SuiTe* evaluations (row) as suitable (S), unsuitable (U), or pending suitability (P), with concordance %.

Caregivers/*SuiTe*	S	P	U	Total	% Concordant
S	9	3	10	22	40.9%
P	0	1	4	5	20%
U	0	1	10	11	90.9%
Total	9	5	24	38	52.6%

## Data Availability

The dataset is available upon request from the corresponding authors. The data are not publicly available due to privacy.

## Supplementary Materials

The following supporting information can be downloaded at: https://www.mdpi.com/article/10.3390/vetsci12121110/s1, File S1, Questionnaire. File S2, Phases and Rationale of Test (*SuiTe*).

## Abbreviations

The following abbreviations are used in this manuscript:

AAIs	Animal-assisted interventions
SuiTe	Suitability test
EIA	Enzyme immunoassay
