# Supplementary material for: A Pre-Screening Tool to Assess Dog Suitability for Animal-Assisted Interventions: Preliminary Results for Dog-Suitability Tests (SuiTe)
Source: Vet Sci. 2025 Nov 22;12(12):1110. doi: 10.3390/vetsci12121110 (PMC12737594; doi:10.3390/vetsci12121110)
Supplement: Supplementary file 1 [file vetsci-12-01110-s001.zip › S1 Supplementary materials (Questionnaire).pdf]

*Supplementary Materials: Questionnaire*

---

Questionario attitudinale sul cane

Il presente questionario è finalizzato ad acquisire maggiori informazioni sul comportamento del cane in diversi contesti e nella relazione con il proprietario, in vista dello svolgimento di test comportamentali.

Il questionario può essere completato da qualsiasi persona maggiorenne che abbia almeno un cane e che sia attivamente coinvolta nella sua gestione. La persona che risponde al questionario deve essere la stessa che condurrà il cane a svolgere i test comportamentali presso il Dip. Scienze Veterinarie – Università di Pisa (per i quali è stato ottenuto parere favorevole dall'Organismo Preposto al Benessere Animale UniPi Delibera n. 02/2025). I dati saranno utilizzati solamente in forma aggregata e nel rispetto della normativa vigente sulla privacy (D. Lgs. 101/2018). Questo studio è stato sviluppato da ricercatori del Dipartimento di Scienze Veterinarie dell'Università di Pisa. Lo studio ha ricevuto parere favorevole del Comitato Bioetico dell'Università di Pisa (delibera n. 16/2025). Per leggere l'informativa sulla privacy visita https://form.jotform.com/250513725033043 o leggi sotto:

* Indica una domanda obbligatoria

Informativa resa ai sensi dell'art. 13 del regolamento UE n. 2016/679

Gentile Signora/e,

desideriamo informarLa che il Regolamento UE n. 679/2016 "Regolamento generale sulla protezione dei dati" prevede la tutela delle persone rispetto al trattamento dei dati personali. Secondo la normativa indicata, tale trattamento sarà improntato ai principi di liceità, correttezza e trasparenza, adeguatezza, pertinenza e limitazione, esattezza e aggiornamento, non eccedenza e responsabilizzazione. I dati personali sono trattati, ai sensi dell'art. 6, comma 1 lettera a) del GDPR, previo Suo consenso. Il trattamento delle categorie particolari di dati personali viene effettuato ai sensi dell'art. 9 comma 2, lettere a) del GDPR.

Pertanto, Le forniamo le seguenti informazioni:

1. I dati da Lei forniti (dati demografici, professione) verranno trattati per finalità di ricerca scientifica, nell'ambito del progetto di ricerca "Valutazione dell'efficacia degli Interventi Assistiti con Animali (IAA) e dello stato di welfare degli animali coinvolti in una logica One Health". La ricerca è finalizzata allo studio della relazione uomo-animale e dei possibili benefici che gli IAA hanno sulle persone e sugli animali. A tal fine ai partecipanti sarà chiesto di svolgere le seguenti attività.

Nell'ambito dello svolgimento di test comportamentali:

- Partecipazione a test attitudinali per valutare l'idoneità del proprio cane per la partecipazione agli IAA;
- Partecipazione a test per valutare il comportamento del cane in presenza e assenza del proprietario;
- Simulazione di visita veterinaria in presenza del proprietario, in cui verrà effettuata anche una raccolta di campioni salivari (attraverso l'utilizzo di tamponi) per le successive analisi endocrinologiche (cortisolo e ossitocina);
- Compilazione di questionari.

I dati saranno raccolti in un'unica sessione, ripetuta due volte a distanza di un mese circa.

2. Il trattamento dei dati personali sarà effettuato con le seguenti modalità. Il titolare del trattamento metterà manualmente in atto misure tecniche e organizzative adeguate, quali la cifratura e la pseudonimizzazione, volte ad attuare in modo efficace i principi di protezione dei dati e a tutelare i diritti degli interessati. I dati saranno conservati per il tempo necessario alle finalità e cancellati dopo cinque anni dalla conclusione programmata della ricerca.

3. La sua partecipazione a questo studio è libera e volontaria. Il conferimento dei dati è obbligatorio e l'eventuale rifiuto di fornire tali dati ha come conseguenza la non partecipazione al progetto di ricerca. Lei potrà inoltre decidere di cambiare la propria opinione dopo aver prestato il consenso, purché lei esprima questa volontà prima che i suoi dati vengano completamente anonimizzati, in quanto a seguito di questo processo non sarà più possibile risalire all'origine dei dati.

4. I dati sanitari saranno comunicati ad altri soggetti (consulenze con medici geriatri e psicologi inclusi nella ricerca) dopo pseudonimizzazione e saranno oggetto di diffusione a scopo di ricerca scientifica sempre in forma anonima e aggregata. I dati anagrafici verranno raccolti da uno dei collaboratori del team di ricerca e saranno oggetto di diffusione a scopo di ricerca scientifica sempre in forma anonima e aggregata.

5. Il Titolare del trattamento è l'Università di Pisa con sede in Pisa, Lungarno Pacinotti 43, nella persona del Rettore pro tempore.
6. Il Responsabile scientifico della ricerca è la Prof.ssa Chiara Mariti, del Dipartimento di Scienze Veterinarie dell'Università di Pisa.
7. Le Persone autorizzate al trattamento dei dati sono: la Prof.ssa Chiara Mariti (Prof.ssa Associata presso il DSV e Responsabile Scientifico del Progetto), il Prof. Francesco Di Iacovo (Vicedirettore del DSV), Prof.ssa Moruzzo (Prof.ssa Associata presso il DSV) e le dottorande presso il DSV Dott.ssa Borrelli, Dott.ssa Granai e Dott.ssa Russo.
8. Presso il Titolare del trattamento è presente il Responsabile della protezione dei dati, nominato ai sensi dell'art. 37 del Regolamento UE 2016/679. Il responsabile della protezione dei dati può essere contattato ai seguenti indirizzi: e-mail: responsabileprotezionedati@unipi.it pec: responsabileprotezionedati@pec.unipi.it.
9. In ogni momento potrà esercitare i Suoi diritti di cui alla sezione 2, 3 e 4 del capo III del Regolamento UE n. 679/2016 (es. diritti di informazione e accesso, di rettifica e cancellazione, di limitazione e di opposizione al trattamento, di portabilità dei dati personali).
10. Lei ha il diritto di presentare un reclamo all'autorità di controllo (in Italia l'Autorità Garante per la protezione dei dati personali, www.garanteprivacy.it, e-mail: protocollo@gpdp.it pec: protocollo@pec.gpdp.it, centralino tel. 06696771).

1.Se hai letto l'Informativa ed acconsenti a partecipare a questo studio, seleziona l'opzione "Sì": *

*Contrassegna solo un ovale.*

Sì
No

## Dati anagrafici

2.Nome e cognome del proprietario *

3.Nome del cane *

## Informazioni proprietario

4.Genere *

*Contrassegna solo un ovale.*

Maschio
Femmina
Preferisco non specificarlo
Altro:

5.Età (indicare il numero in anni, es. 35) *

6.Professione (se più di una, scegli quella che più ti rappresenta) *

*Contrassegna solo un ovale.*

Educatore/Istruttore/Addestratore cinofilo
Veterinario comportamentalista
Veterinario
Altra professione con animali
Studente di corsi di laurea inerenti ad attività con animali (medicina veterinaria, scienze delle produzioni animali, biologia...)
Studente di corsi di laurea non inerenti ad attività con animali
Impiegato
Libero professionista
Disoccupato
Pensionato
Operaio

Altro:

7.Titolo di studio *

*Contrassegna solo un ovale.*

Scuole elementari/medie

Diploma di scuola superiore

Laurea

Post-laurea

8.Nel tuo nucleo familiare, sono presenti altri animali? (più di una risposta possibile) *

*Seleziona tutte le voci applicabili.*

No

Sì, almeno un altro cane

Sì, almeno un gatto

Sì, altri animali diversi da cane/gatto

9.La persona che sta rispondendo al questionario: *

*Contrassegna solo un ovale.*

corrisponde a chi si prende maggiormente cura del cane

si divide la cura del cane insieme a qualcun altr* più o meno in egual misura

non corrisponde a chi si prende cura maggiormente del cane

10.Il tuo cane ha mai partecipato a Interventi Assistiti con Animali IAA (anche conosciuti come "pet-therapy")? *

*Contrassegna solo un ovale.*

No *Passa alla domanda 19.*

Sì, ma non con me *Passa alla domanda 11.*

Sì, con me *Passa alla domanda 11.*

## Interventi Assistiti con Animali (IAA)

Compila questa sezione solo se il cane con cui parteciperai ai test opera/ha operato in IAA.

11.Solitamente il cane con che tipo di utenza lavora?

12.La persona che risponde ha mai svolto la formazione IAA?

*Contrassegna solo un ovale.*

No, non ho svolto la formazione

Sì, sto attualmente svolgendo la formazione

Sì, ho completato il percorso formativo dopo le Linee Guida (2015)

Altro:

13.La persona che risponde ha mai svolto in prima persona IAA? (più di una risposta possibile)

*Seleziona tutte le voci applicabili.*

No

Sì, prima delle Linee Guida (2015)

Sì, sia prima che dopo le Linee Guida (2015)

Sì, dopo le Linee Guida (2015)

Sì e lo faccio tutt'ora

Altro:

14.Sei:

*Contrassegna solo un ovale.*

Sia il proprietario del cane che il suo coadiutore esclusivo

Sia il proprietario del cane che il suo coadiutore, ma talvolta può avere un altro coadiutore
Esclusivamente il coadiutore del cane ma non il proprietario (caregiver)
Solo il proprietario del cane ma non un coadiutore

15.Da quanti anni il tuo cane è coinvolto in IAA? (se è da meno di 1 anno, indicare "< 1 anno", altrimenti indicare il numero di anni ad es. 6)
16.In media quante sessioni di IAA esegue il tuo cane in un mese? (considera un tipico mese in cui il cane lavora a progetti di IAA)

*Contrassegna solo un ovale.*

meno di 4 volte al mese
4 volte al mese
8 volte al mese
12 volte al mese
più di 12 volte al mese
Altro:

17.In media quanto dura una sessione di IAA per il tuo cane?
18.Solitamente come reagisce il cane al trasporto? (più di una risposta possibile)

*Seleziona tutte le voci applicabili.*

Si eccita/stressa in qualsiasi situazione di trasporto
Si eccita/stressa solo quando capisce che deve lavorare (IAA)
Si eccita/stressa quando il percorso prevede molte buche e/o curve
Si eccita/stressa quando capisce che farà qualcosa che gli piace (es. parco)
Si eccita/stressa quando capisce che farà qualcosa che non gli piace (es. veterinario)
È tendenzialmente calmo
Altro:

## Informazioni generali sul cane

19.Età (arrotonda a numero intero, es. 2 anni e 5 mesi = 2 anni; 12 anni e 6 mesi = 13 anni) *
20. Sesso *

*Contrassegna solo un ovale.*

Maschio
Femmina

21.Il tuo cane è stato sterilizzato/castrato? *

*Contrassegna solo un ovale.*

Sì
No

22.Qual è la taglia del tuo cane? *

*Contrassegna solo un ovale.*

Mini (max. 5 kg)
Piccola (6-10kg)
Media (11-25kg)
Grande (26-45kg)
Gigante (>46kg)

23.Razza (se non di razza indicare "meticcio") *

24.Indica la provenienza del tuo cane: *

*Contrassegna solo un ovale.*

Canile/Rifugio

Privato/Allevamento amatoriale
Allevamento professionale
Nato in casa
Negozio di animali
Trovato
Altro:

25.Il tuo cane è attualmente sano? *
*Contrassegna solo un ovale.*
Sì
No

26.Se hai risposto "No" alla domanda precedente, specifica quale/i patologie ha il tuo cane:
27.Nelle ultime due settimane, il tuo cane ha preso farmaci con cortisone? *
*Contrassegna solo un ovale.*
Sì
No

28.Il tuo cane ha avuto patologie a (più di una risposta possibile): *
*Seleziona tutte le voci applicabili.*
Orecchie
Bocca
Zampe
Coda
Nessuna delle precedenti
Altro:

## Risposta all'addestramento

Alcuni cani sono più obbedienti e addestrabili di altri. **Ripensando agli ultimi mesi, indica quanto spesso da 0 (Mai) a 4 (Sempre) il tuo cane ha risposto come descritto in ognuna delle seguenti situazioni:**

29.1. Quando è senza guinzaglio torna subito quando richiamato*
30. 2. Ubbidisce immediatamente al comando "seduto" **
31. 3. Ubbidisce immediatamente al comando "resta" *
32. 4. Sembra prestare attenzione/ascoltare attentamente tutto ciò che dici e/o fai*
33. 5. Risponde lentamente a correzioni o punizioni, ha "la testa dura" *
34. 6. È lento nell'apprendere nuovi esercizi/compiti*
35. 7. È facilmente distratto da rumori, odori o dalla vista di cose interessanti *
36. 8. Riporta o prova a riportare legnetti, palline o altri oggetti **

## Aggressività

Alcuni cani di tanto in tanto esibiscono comportamenti aggressivi. Abbaiare, ringhiare e mostrare i denti sono segni tipici di moderata aggressività. Un'aggressività più grave generalmente include i seguenti comportamenti: scagliarsi contro qualcuno, mordere o tentare di mordere. Ripensando agli ultimi mesi, indica la tendenza del tuo cane a manifestare comportamenti aggressivi, in ciascuna delle situazioni descritte sotto. **Scegli un valore da 0 a 4, dove 0 indica nessun segno di aggressività, e 4 la presenza di segni di aggressività elevata.**
*Contrassegna solo un ovale.*

37. 9. Quando viene corretto o punito verbalmente (rimproverato, sgridato, etc.) da te o da un altro membro del nucleo familiare *

38. 10. Quando viene avvicinato in maniera diretta da una persona adulta sconosciuta, mentre passeggia o fa attività al guinzaglio*
39. 11. Quando viene avvicinato in maniera diretta da un bambino sconosciuto, mentre passeggia o fa attività al guinzaglio*
40.12. Nei confronti di persone sconosciute che si avvicinano al cane quando è in macchina (per esempio, dal benzinaio)
41.13. Quando gli vengono portati via giocattoli, ossi o altri oggetti, da un membro del nucleo familiare*
42.14. Quando viene lavato o spazzolato da un membro del nucleo familiare **
43.15. Quando una persona sconosciuta si avvicina a te o ad un altro membro del nucleo familiare, in casa*
44.16. Quando delle persone sconosciute si avvicinano a te o ad un altro membro del nucleo familiare, fuori casa*
45.17. Quando viene avvicinato in maniera diretta da te o da un altro membro del nucleo familiare, mentre (il cane) sta mangiando*
46.18. Quando un fattorino o il postino si avvicina a casa*
47.19. Quando gli viene portato via il cibo da un membro del nucleo familiare*
48.20. Quando degli estranei passano davanti a casa mentre il cane è fuori casa, in giardino/cortile**
49.21. Quando una persona sconosciuta cerca di toccare o accarezzare il cane*
50.22. Quando ciclisti, gente che fa jogging, persone su skateboard o pattini passano davanti a casa mentre il cane è fuori casa, in cortile/giardino **
51.23. Quando viene avvicinato in maniera diretta da un cane maschio sconosciuto mentre è a passeggio al guinzaglio*
52.24. Quando viene avvicinato in maniera diretta da un cane femmina sconosciuto mentre è a passeggio al guinzaglio. *
53.25. Quando viene guardato fisso negli occhi da un membro del nucleo familiare. * *
54.26. Nei confronti di cani sconosciuti in visita a casa vostra.
55.27. Nei confronti di gatti, scoiattoli o altri animali che entrano nel vostro giardino.
56.28. Nei confronti di persone sconosciute in visita a casa vostra. *
57.29. Quando un cane (sconosciuto) gli abbaia, gli ringhia o gli si scaglia contro. *
58.30. Quando un membro del nucleo familiare lo scavalca/gli passa sopra. *
59.31. Quando tu o un altro membro del nucleo familiare vi riprendete del cibo o degli oggetti precedentemente rubati dal cane. *
60.31bis. Ci sono altre situazioni in cui il tuo cane mostra, a volte, un comportamento aggressivo?

*Contrassegna solo un ovale.*

Sì

No

Non so

61.32. Nei confronti di un altro cane (conosciuto) che vive nella stessa casa (lasciare vuoto se non ci sono altri cani in casa). *
62.33. Quando viene avvicinato da un altro cane (conosciuto) che vive nella stessa casa mentre si trova nella sua cuccia/luogo di riposo preferito/a (lasciare vuoto se non ci sono altri cani in casa). *
63.34. Quando viene avvicinato da un altro cane (conosciuto) che vive nella stessa casa, mentre sta mangiando (lasciare vuoto se non ci sono altri cani in casa). *
64.35. Quando viene avvicinato da un altro cane (conosciuto) che vive nella stessa casa mentre mastica/gioca con un giocattolo preferito, osso, oggetto, etc. (lasciare vuoto se non ci sono altri cani in casa). *

## Paura e ansia

I cani a volte mostrano segni di ansia o di paura, quando sono esposti a rumori, oggetti, persone o situazioni particolari. Tipici segni di lieve o moderata paura includono i seguenti comportamenti: evitare lo sguardo, evitare l'oggetto temuto, appiattirsi a terra o rannicchiarsi con la coda bassa o tra le zampe, uggiolare o guaire, immobilizzarsi e tremare. Una paura estrema è caratterizzata da comportamenti quali: appiattirsi o rannicchiarsi in maniera esagerata, effettuare tentativi vigorosi di fuga, allontanarsi o nascondersi dall'oggetto, dalla persona o dalla situazione temuti. Ripensando agli ultimi mesi, indica la tendenza del tuo cane a mostrare paura o ansia

nelle situazioni descritte in seguito. **Scegli un valore da 0 a 4, dove 0 indica nessun segno di paura/ansia, e 4 indica un livello estremo di paura/ansia.**

*Contrassegna solo un ovale.*

65.36. Quando viene avvicinato in maniera diretta da una persona adulta sconosciuta, non in casa*
66.37. Quando viene avvicinato in maniera diretta da un bambino sconosciuto, fuori casa*
67.38. In risposta a rumori improvvisi o forti (ad esempio: aspirapolvere, rombi di motori, martello pneumatico, oggetti che cadono accidentalmente) *
68.39. Quando delle persone sconosciute vengono a casa vostra
69.40. Quando una persona sconosciuta cerca di toccarlo o accarezzarlo*
70.41. In situazioni di traffico auto intenso.
71.42. In risposta a oggetti strani e poco familiari, che si trovano sul marciapiedi o vicino ad esso (ad esempio sacchetti della spazzatura, foglie, cartacce, bandiere sventolanti, etc.) **
72.43. Quando viene visitato/medicato dal veterinario**
73.44. Durante temporali, fuochi d'artificio o eventi simili**
74.45. Quando viene avvicinato in maniera diretta da un cane sconosciuto della stessa taglia o più grande. *
75.46. Quando viene avvicinato in maniera diretta da un cane di taglia più piccola*
76.47. Quando si è trovato, per la prima volta, in situazioni sconosciute (per esempio: il primo viaggio in macchina, la prima volta in ascensore, la prima visita dal veterinario). * *
77.48. Quando c'è vento o ci sono oggetti mossi dal vento*
78.49. Quando gli vengono tagliate le unghie da un membro del nucleo familiare.
79.50. Quando viene spazzolato o lavato da un membro del nucleo familiare*
80.51. Quando gli vengono asciugate le zampe da un membro del nucleo familiare*
81.52. Quando cani sconosciuti vengono a casa vostra
82.53. Quando un cane sconosciuto gli abbaia, gli ringhia o gli si scaglia contro*

## Comportamenti relativi a momenti di separazione

Alcuni cani mostrano segni di ansia o altri comportamenti problematici, quando vengono lasciati soli, anche per periodi relativamente brevi. **Ripensando agli ultimi mesi, indica quanto spesso da 0 (Mai) a 4 (Sempre) il tuo cane ha risposto come descritto nei seguenti punti, quando è stato lasciato solo, o appena prima di essere lasciato solo.**

*Contrassegna solo un ovale.*

83.54. Scuotersi, rabbrividire, tremare*
84.55. Salivazione eccessiva*
85.56. Irrequietezza/agitazione/camminare incessantemente*
86.57. Uggiolare *
87.58. Abbaiare*
88.59. Ululare*
89.60. Rosicchiare/grattare porte, finestre, pavimenti, tende, etc. **
90.61. Perdita di appetito*
91.61. bis Ci sono altre situazioni in cui il tuo cane ha paura o è ansioso? *

*Contrassegna solo un ovale.*

Sì
No
Non so
Altro:

## Eccitabilità

Alcuni cani non mostrano reazioni evidenti a eventi improvvisi o potenzialmente eccitanti, né a cambiamenti nel loro ambiente; altri invece si eccitano molto alla più piccola novità. I seguenti comportamenti sono segni di una eccitabilità da lieve a moderata: allerta, dirigersi verso la situazione/oggetto nuovo, brevi episodi di abbaio. L'estrema eccitabilità è caratterizzata da una generale tendenza a reagire in maniera esagerata. Il cane

eccitabile abbaia o guaisce in modo isterico al più piccolo cambiamento, corre incontro o tutto attorno a quel che lo eccita ed è difficile da calmare. Ripensando agli ultimi mesi, indica la tendenza del tuo cane a mostrare un comportamento eccitabile nelle circostanze descritte in seguito. **Scegli un valore da 0 a 4, dove 0 indica un cane calmo, e 4 un cane estremamente eccitabile.**

*Contrassegna solo un ovale.*

92.62. Quando tu o un altro membro del nucleo familiare tornate a casa dopo un breve periodo di assenza * *
93.63. Quando gioca con te o altri membri del nucleo familiare**
94.64. Quando suona il citofono/campanello*
95.65. Appena prima di essere portato fuori per una passeggiata*
96.66. Appena prima di essere portato in giro in macchina
97.67. Quando arriva gente a casa**
98.67bis. Ci sono altre situazioni in cui il tuo cane, a volte, diventa eccessivamente agitato? *

*Contrassegna solo un ovale.*

Si
No
Non so
Altro:

## Attaccamento e ricerca di attenzione

La maggior parte dei cani è fortemente attaccata al loro proprietario, e alcuni di loro richiedono una grande quantità di attenzioni e affetto. **Ripensando all'ultimo periodo, indica quanto spesso da 0 (Mai) a 4 (Sempre) il tuo cane ha mostrato ognuno dei seguenti segni di attaccamento o richiesta di attenzione**.

*Contrassegna solo un ovale.*

99.68. Mostra un forte attaccamento verso uno specifico membro della famiglia. *
100.69. Tende a seguirti (o altri membri del nucleo familiare) in giro per casa, di stanza in stanza. *
101.70. Tende a sedersi vicino o a contatto con te (o altri) quando siete seduti. *
102.71. Tende a dare colpetti con il naso o a dare zampate per ottenere attenzione da te (o altri) quando siete seduti. *
103.72. Si agita (uggiola, salta addosso, cerca di mettersi in mezzo) se tu (o altri) mostrate affetto per un'altra persona. *
104.73. Si agita (uggiola, salta addosso, cerca di mettersi in mezzo) quando tu (o altri) mostrate affetto per un altro cane/animale. *

## Altri comportamenti

I cani mostrano un'ampia gamma di problemi comportamentali oltre a quelli precedentemente menzionati nel questionario. **Ripensando all'ultimo periodo, indica quanto spesso da 0 (Mai) a 4 (Sempre) il tuo cane ha mostrato ognuno dei seguenti comportamenti:**

*Contrassegna solo un ovale.*

105.74. Rincorre i gatti, o li rincorrerebbe se ne avesse la possibilità. *
106.75. Rincorre gli uccelli, o li rincorrerebbe se ne avesse la possibilità. *
107.76. Rincorre gli scoiattoli o li rincorrerebbe se ne avesse la possibilità. *
108.77. Scappa o scapperebbe da casa, o dal giardino/cortile (se ne avesse la possibilità). *
109.78. Si rotola nelle feci di animali o in altre sostanze "puzzolenti". * *
110.79. Mangia i propri escrementi o bisogni, o quelli di altri animali. * *
111.80. Rosicchia oggetti inappropriati. *
112.81. "Monta" oggetti, mobili o persone. * *
113.82. Elemosina cibo in continuazione quando la gente mangia. * *
114.83. Ruba cibo. * *
115.84. È ansioso o ha paura di scendere e/o salire le scale. * *

116.85. Tira eccessivamente quando è al guinzaglio. * *
117.86. Urina su mobili o altri oggetti in casa. *
118.87. Urina quando viene avvicinato, accarezzato, manipolato o preso in braccio. * *
119.88. Urina se lasciato solo di notte o di giorno. *
120.89. Defeca se lasciato solo di notte o di giorno. *
121.90. È iperattivo e irrequieto, ha difficoltà a calmarsi. * *
122.91. Giocherellone, cucciolone, esuberante. *
123.92. Attivo, energico, deve sempre fare qualcosa. *
124.93. Tiene lo sguardo fisso nel vuoto. * *
125.94. Sembra voler catturare le mosche (invisibili). * *
126.95. Si rincorre la coda/il posteriore. * *
127.96. Rincorre/insegue le ombre o i punti luminosi. * *
128.97. Abbaia ostinatamente quando allarmato o eccitato. * *
129.98. Si lecca eccessivamente. * *
130.99. Lecca oggetti o persone eccessivamente. * *
131.100. Mostra altri comportamenti bizzarri, strani o ripetitivi. * *

## Adulti e oggetti sconosciuti

132.Mostra Paura e Ansia/Aggressività/Eccitabilità quando incontra una persona adulta sconosciuta, non in casa, vestita in modo inusuale (es: camice bianco, mascherina, occhiali da sole, cappello, uso di bastone/stampella)? *

*Seleziona tutte le voci applicabili.*

No
Sì, Paura e Ansia
Sì, Aggressività
Sì, Eccitabilità

133.Mostra Paura e Ansia/Aggressività/Eccitabilità quando incontra un gruppo di persone sconosciute, non in casa? *

*Seleziona tutte le voci applicabili.*

No
Sì, Paura e Ansia
Sì, Aggressività
Sì, Eccitabilità

134.Mostra Paura e Ansia/Aggressività/Eccitabilità quando viene accarezzato da un gruppo di persone sconosciute, non in casa? *

*Seleziona tutte le voci applicabili.*

No
Sì, Paura e Ansia
Sì, Aggressività
Sì, Eccitabilità

135.Mostra Paura e Ansia/Aggressività/Eccitabilità quando passa davanti a oggetti in movimento come, per esempio, biciclette, sedie a rotelle, carrelli? *

*Seleziona tutte le voci applicabili.*

No
Sì, Paura e Ansia
Sì, Aggressività
Sì, Eccitabilità

136.Mostra Paura e Ansia/Aggressività/Eccitabilità quando viene avvicinato in maniera diretta da una persona adulta sconosciuta che lo/a invita a giocare? *

*Seleziona tutte le voci applicabili.*

No
Sì, Paura e Ansia
Sì, Aggressività
Sì, Eccitabilità

137.Mostra Paura e Ansia/Aggressività/Eccitabilità quando viene spazzolato da una persona adulta sconosciuta (es. toelettatore)? *

*Seleziona tutte le voci applicabili.*

No
Sì, Paura e Ansia
Sì, Aggressività
Sì, Eccitabilità

138.Secondo te il tuo cane sarebbe adatto a svolgere Interventi Assistiti con Animali (anche conosciuti come pet-therapy)? Rispondi con Sì/No e con il perchè della tua risposta. *
