# Supplementary material for: A Pre-Screening Tool to Assess Dog Suitability for Animal-Assisted Interventions: Preliminary Results for Dog-Suitability Tests (SuiTe)
Source: Vet Sci. 2025 Nov 22;12(12):1110. doi: 10.3390/vetsci12121110 (PMC12737594; doi:10.3390/vetsci12121110)
Supplement: Supplementary file 1 [file vetsci-12-01110-s001.zip › S2 Supplementary materials (Phases and Rationale of Test).pdf]

Subphases	Sources	Rationale for Inclusion	References for Rationale
Room exploration	modified from PADA protocol [11]	Absence of phobias and neophobia, as the fear of (novel) objects or situations constrains exploratory behaviour and limits opportunities for learning.	Glenk & Foltin, 2021 [7]
Food and Toy motivation	modified from Rapporto ISTISAN 19/4 [3]	Risk of bites, falls, or scratches to participants if the dog is too excited. Food and toys are often used to engage the users (e.g. dog training exercises).	Meers et al., 2022 [28]; Francia et al., 2019 [3]
Unusual static/moving objects	modified from PADA protocol [11]	Common objects which can be present in different AAI settings (e.g. schools, prisons, elderly homes).	Glenk, 2017 [4]; Clark et al., 2020 [21]
Sounds	Authors' previous experience in the field of AAIs	Possible exposure to high levels of noise during AAIs.	Glenk & Foltin, 2021 [7]
Separation	modified from Rapporto ISTISAN 19/4 [3] and authors' previous experience	Dogs should be comfortable being left alone under different circumstances.	Winkle et al., 2020 [22]
Social attraction (familiar and unfamiliar people)	modified from Rapporto ISTISAN 19/4 [3] and PADA protocol [11]	A suitable dog should exhibit prosociality	Francia et al., 2019 [3]
Handling (familiar and unfamiliar people) and Brush	modified from Mongillo et al., 2014 [9] and authors' previous experience	Suitable dogs are expected to tolerate physical intimacy with strangers and to remain there calmly	Glenk, 2017 [4]
Group	modified from Mongillo et al., 2014 [9] and PADA protocol [11]	AAIs could be single/group sessions	Glenk & Foltin, 2021 [7]
White coat and surgical face mask	modified from Mongillo et al., 2014 [9]	AAIs can take place in hospital settings	Glenk & Foltin, 2021 [7]
Crutches and sunglasses	modified from PADA protocol [11], Mongillo et al., 2014 [9] and authors' previous experience	AAI can involve individuals using crutches or wearing sunglasses	Mongillo et al., 2014 [9]
Baby doll	Authors' previous experience	Indirect evidence of a child's presence, which may occur during AAIs in schools or hospitals	Glenk & Foltin, 2021 [7]
Training and concentration	modified from Rapporto ISTISAN 19/4 [3] and Mongillo et al., 2014 [9]	The dog may be distracted during the AAI session	Glenk, 2017 [4]; Mongillo et al., 2014 [9]

S2: Supplementary materials (Phase and Rationale of SuiTe). Table with Subphases of SuiTe; Sources from which the subphases were modified and inserted in SuiTe; the Rationale for Inclusion of the Suphases; References for the Rationale.
